# Diaphragm weakness in mechanically ventilated critically ill patients

**DOI:** 10.1186/cc12792

**Published:** 2013-06-20

**Authors:** Gerald S Supinski, Leigh Ann Callahan

**Affiliations:** 1 Department of Internal Medicine, Division of Pulmonary, Critical Care and Sleep Medicine, University of Kentucky, 740 South Limestone Room L-543, Lexington, KY 40536-0284, USA

## Abstract

**Introduction:**

Studies indicate that mechanically ventilated patients develop significant diaphragm muscle weakness, but the etiology of weakness and its clinical impact remain incompletely understood. We assessed diaphragm strength in mechanically ventilated medical ICU patients, correlated the development of diaphragm weakness with multiple clinical parameters, and examined the relationship between the level of diaphragm weakness and patient outcomes.

**Methods:**

Transdiaphragmatic twitch pressure (PdiTw) in response to bilateral magnetic stimulation of the phrenic nerves was measured. Diaphragm weakness was correlated with the presence of infection, blood urea nitrogen, albumin, and glucose levels. The relationship of diaphragm strength to patient outcomes, including mortality and the duration of mechanical ventilation for successfully weaned patients, was also assessed.

**Results:**

We found that infection is a major risk factor for diaphragm weakness in mechanically ventilated medical ICU patients. Outcomes for patients with severe diaphragm weakness (PdiTw <10 cmH_2_O) were poor, with a markedly increased mortality (49%) compared to patients with PdiTw ≥10 cmH_2_O (7% mortality, *P *= 0.022). In addition, survivors with PdiTw <10 cmH_2_O required a significantly longer duration of mechanical ventilation (12.3 ± 1.7 days) than those with PdiTw ≥10 cmH_2_O (5.5 ± 2.0 days, *P *= 0.016).

**Conclusions:**

Infection is a major cause of severe diaphragm weakness in mechanically ventilated patients. Moreover, diaphragm weakness is an important determinant of poor outcomes in this patient population.

## Introduction

The number of mechanically ventilated patients in medical ICUs (MICUs) in the United States has increased dramatically over the past 20 years. Currently 800,000 patients per year require mechanical ventilation [1]. Many of these patients die, with an annual mortality exceeding 200,000 [2]. In addition, survivors often require prolonged, expensive hospital stays to achieve liberation from mechanical ventilation [3]. In the past it was thought that the severity of lung disease was the major determinant of outcomes in MICU patients, but recent work indicates that mechanically ventilated patients develop significant diaphragm weakness [4-6].

Diaphragm weakness is primarily thought to occur as a consequence of ventilator-induced diaphragm inactivity, with weakness progressing as duration of mechanical ventilation increases [7,8]. Theoretically, however, there are other mechanisms by which diaphragm weakness can develop. Animal studies indicate that experimental models of infection induce significant diaphragm weakness [9,10]. In addition, data suggest that azotemia, hyperglycemia, and low systemic albumin levels are risk factors for prolonged mechanical ventilation and could theoretically be associated with the development of respiratory muscle weakness [11-13]. The importance of infection, azotemia, hyperglycemia, and reduced albumin levels as risk factors for the development of diaphragm weakness in mechanically ventilated patients, however, is unknown.

Diaphragm weakness is also commonly thought to predispose patients to sustained respiratory failure, greatly prolonging the time required to wean patients from mechanical ventilation and worsening clinical outcomes. No previous study, however, has examined the quantitative relationship of diaphragm function, assessed using a purely objective, nonvolitional technique (such as bilateral anterior magnetic phrenic nerve stimulation), to clinical outcomes in mechanically ventilated patients.

The purpose of the present study, therefore, was to objectively measure diaphragm strength in a cross-section of mechanically ventilated MICU patients and to test the specific hypothesis that the severity of diaphragm weakness would correlate with one or more of the following clinical factors: the presence of infection, blood urea nitrogen level, serum albumin level, and/or blood glucose level. We also ascertained the relationship of diaphragm strength to patient outcomes, including mortality, rate of transfer to long-term acute care (LTAC) facilities, and the subsequent duration of mechanical ventilation in MICU survivors who were successfully extubated. Finally, to determine whether clinicians were cognizant of the severity of diaphragm weakness present in their patients, we asked the attending MICU physicians to estimate diaphragm strength and compared these estimates with objectively determined measurements.

## Methods

### Study protocol

Studies were performed on adult ICU patients requiring mechanical ventilation in the University of Kentucky MICU for more than 24 hours. The protocol was approved by the University of Kentucky Institutional Review Board and informed consent was obtained from subjects and their surrogates. The following were recorded: diaphragm strength by measuring the transdiaphragmatic twitch pressure (PdiTw); respiratory static system compliance and airway resistance using the mechanical ventilator diagnostic module; basic clinical data; clinician estimates of diaphragm strength; and outcomes, including mortality, rate of transfer to LTAC facilities, and additional days required for continued mechanical ventilation until successful extubation.

### Exclusion criteria

If the attending physician anticipated that the patient would be successfully weaned from mechanical ventilation in less than 24 hours, or determined that the patient was too unstable to tolerate the measurements, subjects were not screened for study inclusion. Exclusion criteria included: requirement for high dose pressors (≥15 μg/minute norepinephrine or ≥15 mg/kg/minute dopamine); elevated positive end-expiratory pressure (PEEP ≥15 cmH_2_O); presence of a cardiac pacemaker or implanted defibrillator; administration of neuromuscular blocking agents within 48 hours prior to study entry; recent variceal bleeding; pregnancy; incarceration; or institutionalization.

### Determination of transdiaphragmatic twitch pressure

Diaphragm strength was assessed by measuring PdiTw in response to bilateral anterior magnetic stimulation of the phrenic nerves. PdiTw is an objective, nonvolitional technique that has been verified in previous studies to provide the most accurate assessment of diaphragm strength in humans [14-16]. Moreover, previous studies demonstrate that this technique can reliably and reproducibly measure diaphragm contractile strength in mechanically ventilated ICU patients [4-6].

Subjects were studied in the supine position with the head of the bed elevated at 30°. Two sterile commercially available balloon-tipped catheters (Ackrad Laboratories, NJ, USA) were passed through the nose after application of local anesthetic (1 milliliter of 1% Lidocaine gel); one catheter was placed in the stomach, while the other was placed in the esophagus. Following initial placement, the catheters were connected to Validyne pressure transducers (Validyne Engineering, Northridge, CA, USA) to verify correct positioning. Correct placement of the gastric balloon was confirmed by demonstrating a positive pressure in response to pressure applied over the stomach; correct placement of the esophageal balloon was verified by demonstrating that the pressure waveform had an end-expiratory pressure similar to the total PEEP level and also mirrored airway pressure changes with inspiratory efforts during airway occlusion.

After confirming accurate balloon placement, subjects were left to breathe quietly for 10 minutes before further assessment. Figure of eight magnetic coils attached to dual Magstim 200 stimulators (Jali Medical, Inc., Waltham, MA, USA) were then placed bilaterally over the phrenic nerves adjacent to the border of the sternocleidomastoid muscles. Magnetic field strength was adjusted to maximal levels (100%) and simultaneous supramaximal magnetic pulses were delivered to the phrenic nerves bilaterally to elicit maximal twitch transdiaphragmatic pressures (that is, PdiTw). Stimuli were interpolated between adjacent ventilator breaths and the transdiaphragmatic pressures elicited by these stimuli were recorded while simultaneously and transiently occluding the external circuit connecting the endotracheal tube to the ventilator with a pneumatic valve. A minimum of five twitches were recorded, with at least 30 seconds between adjacent stimuli. To verify stimuli were supramaximal, additional twitches were performed at reduced magnetic field strengths (90 to 95%). PdiTw was calculated as follows:

pdiTw=Δgastricpressure-Δesophagealpressure

The best three measurements in response to 100% levels of magnetic stimulation were averaged for each subject and recorded as the PdiTw.

### Measurement of respiratory system static compliance and airway resistance

For these assessments, the ventilator was set to a square-wave flow pattern with an inspiratory plateau. The ventilator rate was then transiently increased (for example, 30 to 60 seconds) to suppress spontaneous respirations. After reaching a steady state, the peak pressure and the plateau pressure were recorded and intrinsic PEEP was determined using an end-expiratory occlusion maneuver. Inspiratory airway resistance of the respiratory system was calculated as:

$$\left(\text{Peak}\;\text{pressure - plateau}\;\text{pressure}\right)/)\text{inspiratory}\;\text{flow}$$

The effective static compliance of the respiratory system was calculated as:

$$\text{Tidal}\;\text{volume}/)\left(\text{plateau}\;\text{pressure}-\left(\text{total}\;\text{PEEP}\right)\right)$$

Once measurements were completed, the ventilator was returned to its previous mode and settings.

### Clinical parameters

Data for the following clinical parameters were collected as close as possible to the time of determination of PdiTw levels: age, gender, clinical diagnoses, the presence of positive cultures for infectious agents, antibiotic regimen, glucose, albumin, blood urea nitrogen (BUN), Sequential Organ Failure Assessment scores, Charlson comorbidity indexes, vital signs, duration of mechanical ventilation prior to PdiTw measurement, mechanical ventilation mode, FiO_2 _**(fraction of inspired oxygen)**, tidal volume and rate, percentage of patient triggered breaths, and most recent arterial blood gas values. All recorded values were obtained within 24 hours of PdiTw assessment.

### Clinician estimates

Attending physicians were asked to estimate the level of diaphragm strength using a form with qualitative descriptors of muscle weakness (see Additional file 1).

### Statistical analysis

Whenever data were normally distributed and variances were similar, parametric tests were used to compare groups. When these conditions were not met, nonparametric tests were used to make comparisons. Data analyzed using parametric tests are presented as mean ± standard error of the mean. Data analyzed using nonparametric tests are presented as median ± confidence intervals. Linear regression was utilized to assess the relationship of BUN, albumin, glucose and duration of prior mechanical ventilation to PdiTw level. Analysis of variance was employed to compare PdiTw across cohorts of patients with different levels of ventilator triggering. Fisher exact testing and receiver operating curve analyses were used to determine the boundary between weak and strong PdiTw groups that best discriminated between survival and mortality [17].

## Results

### Diaphragm strength in medical ICU patients

**Figure 1:**
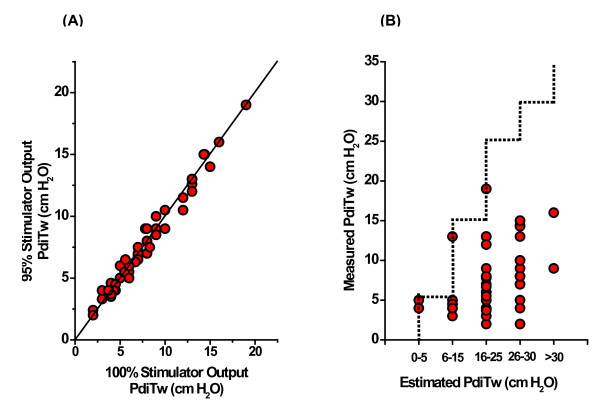
**Transdiaphragmatic twitch pressure: measured levels and physician estimates**. **(A) **Transdiaphragmatic twitch pressure (PdiTw) levels for the 57 subjects included in the analysis. Each symbol represents a single subject and plots the PdiTw level obtained in response to stimulation of the phrenic nerves with 95% of maximum magnetic field strength (*y *axis) against the PdiTw obtained in response to stimulation of the phrenic nerves with 100% of maximum magnetic field strength (*x *axis). All data cluster along the line of identity, indicating that supramaximal stimulation was achieved during 100% magnetic field stimulation. If supramaximal conditions had not been achieved, data points would have fallen to the right of the line of identity. **(B) **Measured PdiTw levels compared with levels predicted for each subject by their attending physicians; each symbol represents data from a single patient. Red symbols below the line (46 out of 51 determinations) indicate determinations for which physicians overestimated diaphragm strength (that is, PdiTw).

Sixty subjects were recruited into the study. PdiTw could not be measured in three subjects because the magnetic coils could not be effectively positioned due to anatomic constraints (Subjects 18, 43, and 48). Detailed information for the 57 subjects in whom PdiTw measurements were successfully performed is provided in Additional file 2. To verify that we achieved supramaximal levels of magnetic stimulation, we plotted the PdiTw values achieved using 95% magnetic field strength levels against PdiTw values attained using 100% magnetic field strength, as shown in Figure 1A. The PdiTw levels obtained using 95% and 100% field strength levels were virtually identical, arguing that supramaximal neural stimulation was achieved when employing 100% magnetic field strength for these studies. Moreover, the twitch determinations were highly reproducible in individual subjects, with a coefficient of variation for the best three measurements performed at 100% stimulator output averaging 7% for the 57 subjects. High levels of PEEP can alter the relationship between the actual intrinsic diaphragm strength and the measured PdiTw. In the present cohort of patients, however, only one study subject had PEEP >8 cmH_2_O. As a result, PEEP-induced hyperinflation did not appreciably impact our data analysis (see Additional file 3).

This cohort of 57 mechanically ventilated subjects had a mean PdiTw of 7.9 ± 0.6 cmH_2_O. This value is similar to values reported previously in mechanically ventilated critically ill patients [4-6]. For comparison, normal healthy adults average a PdiTw of 29.3 ± 2.8 cmH_2_O in our laboratory; this value is similar to that reported for healthy adults in the literature [4,14]. Estimates of diaphragm strength from the attending physicians were obtained for 51 subjects. Clinicians did not accurately predict the level of diaphragm strength of their patients (Figure 1B). In many cases, patients with profound levels of diaphragm weakness were thought to have normal strength. Strength was overestimated in 46 of 51 patients, was correctly estimated in five patients, and was never underestimated.

### Risk factors for the development of diaphragm weakness

**Figure 2:**
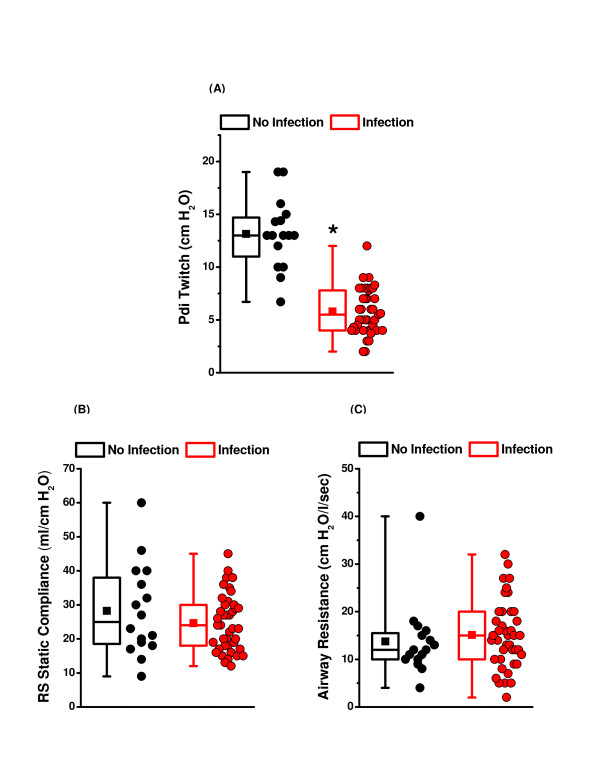
**Infection and diaphragm weakness**. **(A) **Transdiaphragmatic twitch pressure (PdiTw) measurements for non-infected and infected patients. Data from individual patients are shown for each group on the right, while plots on the left for each group show mean (filled squares), median levels (middle line of box), 25% and 75% confidence intervals (upper and lower borders of the box) and 1% and 99% intervals (whiskers above and below the box). Infection was associated with significant lower Pdi Twitch values (*statistical significance). **(B) **respiratory system (RS) static compliance and **(C) **inspiratory airway resistance for non-infected and infected patients; there was no difference in these indices of lung function between non-infected and infected groups.

Data were analyzed to determine which factors correlated with the level of diaphragm weakness in mechanically ventilated subjects. We found a strong relationship between the presence of infection and diaphragm weakness. In all, 41 subjects were classified as being infected based on a positive test for a pathogenic organism from a sterile site (40 patients) or a clinical diagnosis of bacterial pneumonia (one patient; cultures were lost for this individual). All 41 subjects classified as infected were thought by the attending physicians who were providing care for these patients to be infected clinically and all 41 of these patients received antibiotic therapy (Tables 2 and 3). The remaining 16 patients were classified as non-infected (see Table 2). Infected patients had a median PdiTw of only 5.5 cmH_2_O (25 to 75% confidence levels of 4.0 to 7.9 cmH_2_O), while patients without clinical evidence of infection had a median PdiTw of 13.0 cmH_2_O (25 to 75% confidence levels of 11.0 to 14.7, *P *< 0.001) (Figure 2A). Of interest, while infection was associated with greater diaphragm weakness, infected patients did not have significantly different respiratory mechanical parameters (that is, respiratory system static compliance and airway resistance) from non-infected patients (Figure 2B, C).

**Table 1:** Characteristics of non-infected and infected study subjects

	Non-infected (*n *= 16)	Infected (*n *= 41)
Age (years)	52.4 ± 14.1	55.5 ± 16.7
Gender (%)		
Male	44	49
Female	56	51
Body mass index	30.3 ± 9.2	29.8 ± 9.5
Total ICU days	15.1 ± 9.8	34.9 ± 40.2
Days of MV before PdiTw measurement	9.1 ± 8.9	10.4 ± 12.4
Steroid usage (%)	50	51

Data presented as mean ± standard deviation. MV, mechanical ventilation; PdiTw, transdiaphragmatic twitch pressure.

**Table 2:** Classification of subjects according to presence/absence of active infection at time of PdiTw measurements^a^

Subject number	Infected	Site	Organism(s) isolated from site(s)	Diagnosis of infection by attending physician	Antibiotic therapy^b^	Pulmonary infiltrates
1	No			No	No	No
2	Yes	Liver abscess	*Staphylococcus *sp., *Fusobacterium necrophorum*	Yes	Yes	Yes
3	No			No	No	No
4	Yes	PAL	*Streptococcus pneumonia*	Yes	Yes	Yes
5	No			No	No	No
6	Yes	Blood	Gram-positive bacteria^c^	Yes	Yes	Yes
7	Yes	PAL	*Pseudomonas aeruginosa*	Yes	Yes	Yes
8	Yes	Blood	*Staphylococcus species*	Yes	Yes	Yes
9	No			No	No	Yes^d^
10	Yes	PAL	*Pseudomonas aeruginosa*	Yes	Yes	Yes
11	No			No	No	No
12	Yes	Sinuses	*Bacteroides capillosus, Fusobacterium *sp., β-strep. Gp C	Yes	Yes	Yes
		Nasopharyngeal swab	Influenza A			
13	No			No	No	No
14	Yes	Jejunal drain	VRE, *Pseudomonas aeruginosa*	Yes	Yes	Yes
15	Yes	Blood	*Staphylococcus aureus*	Yes	Yes	No
16	No			No	No	No
17	Yes	PAL	*Staphylococcus *species	Yes	Yes	Yes
		Blood	*Staphylococcus *species			
19	Yes	PAL	*Klebsiella pneumonia*	Yes	Yes	Yes
20	Yes	Blood	*Staphylococcus aureus*	Yes	Yes	No
21	Yes	Neck abscess	*Streptococcus *sp.	Yes	Yes	No
22	Yes	Sputum	*Hemophilus parainfluenza*	Yes	Yes	Yes
23	Yes	Stage IV decubitus	*Pseudomonas aeruginosa*, *Proteus mirabilis*	Yes	Yes	No
		Urine	*Pseudomonas aeruginosa*			
24	No			No	No	No
25	No			No	No	No
26	No			No	No	No
27	Yes	Subphrenic abscess	*Candida glabrata*	Yes	Yes	Yes
28	No			No	No	No
29	Yes	BAL	*Escherichia coli*, *Klebsiella pneumoniae*	Yes	Yes	Yes
30	No			No	No	No
31	No			No	No	Yes^d^
32	Yes	Blood	*Enterococcus faecalis*	Yes	Yes	No
33	Yes	PAL	*Staphylococcus aureus*	Yes	Yes	Yes
34	Yes	Blood	Gram-positive cocci^c^	Yes	Yes	Yes
35	Yes	ET aspirate	*Acinetobacter calcoaceticus*, *Enterobacter cloacae*	Yes	Yes	Yes
		Blood	*Candida*			
36	Yes	PAL	*Achromobacter xyloxosidans*	Yes	Yes	Yes
		Central venous catheter	*Pseudomonas aeruginosa*			
37	Yes	PAL	*Pseudomonas aeruginosa*	Yes	Yes	Yes
38	Yes	Liver abscess	*Escherichia coli*	Yes	Yes	Yes
39	Yes	PAL	*Staphylococcus *sp., *Enterobacter aerogenes*, *Escherichia coli*	Yes	Yes	Yes
40	Yes	PAL	MRSA	Yes	Yes	Yes
		Blood	MRSA			
41	Yes	Nasopharyngeal swab	Influenza B	Yes	Yes	Yes
42	Yes	Blood	MRSA	Yes	Yes	Yes
		Leg abscess	MRSA			
		Osteomyelitis	MRSA			
44	No			No	No	Yes^d^
45	No			No	No	No
46	Yes	Sputum	MRSA	Yes	Yes	Yes
47	Yes	PAL	*Streptococcus pneumonia*	Yes	Yes	Yes
49	No			No	No	No
50	Yes	Pleural fluid	*Acinetobacter baumannii*, VRE	Yes	Yes	Yes
		Pleural tissue	*Acinetobacter baumannii*, VRE			
51	Yes	Blood	*Bacillus circulans*	Yes	Yes	Yes
52	Yes	Blood	VRE	Yes	Yes	Yes
53	Yes	PAL	*Pseudomonas aeruginosa*	Yes	Yes	Yes
		Nasopharyngeal swab	H1N1			
54	Yes	Blood	VRE	Yes	Yes	Yes
		Tracheal aspirate	*Stenotrophomonas maltophilia*			
55	Yes	PAL	MRSA	Yes	Yes	Yes
56	Yes	Blood	MRSA, *Pseudomonas aeruginosa*, VRE	Yes	Yes	Yes
		Sputum	*Pseudomonas aeruginosa*			
57	Yes	BAL	Samples lost^e^	Yes	Yes	Yes
58	Yes	Tracheal aspirate	*Hemophilus influenza*	Yes	Yes	Yes
59	Yes	Blood	*Staphylococcus species*	Yes	Yes	No
60	Yes	Urine	*Enterobacter cloacae*	Yes	Yes	Yes
		PAL	*Streptococcus *sp.			

^a^Data not included for Subjects 18, 43, and 48 because transdiaphragmatic twitch pressure (PdiTw) measurements were not obtained due to anatomic constraints. ^b^Patient on antibiotic therapy at the time of PdiTw measurement. ^c^Organism not speciated - subject treated with antibiotics prior to transfer.^d^Patient with pulmonary infiltrates but without evidence of infection (Subject 9 with nonpulmonary acute lung injury, Subject 31 with pulmonary fibrosis, Subject 44 with acute respiratory distress syndrome). ^e^Bronchoscopy performed with purulent exudates noted; specimens lost in transit to laboratory. BAL, bronchoalveolar lavage; MRSA, methicillin-resistant *Staphylococcus aureus*; PAL, protected alveolar lavage; VRE, *Enterococcus faecium *(vancomycin-resistant).

**Table 3:** Medication regimen in 57 subjects at the time of transdiaphragmatic twitch pressure measurements^a^

Subject number	Antibiotics	Other medications	Steroid regimen over entire ICU stay
1		Midazolam, metoprolol, hydralazine, calcium acetate, famotidine	
2	Piperacillin/tazobactam, vancomycin, levofloxacin, flagyl	Enoxaparin, omeprazole, dobutamine	Hydrocortisone 100 mg every 8 hours × 4 days
3		Metoprolol, haloperidol, insulin, aspirin	
4	Piperacillin/tazobactam, vancomycin, levofloxacin	Midazolam, fentanyl, pantoprazole	
5		Famotidine, trazadone	Methylprednisolone 1 g/day × 3 days, prednisone 60 mg/day × 2 days, 40 mg/day × 3 days, 30 mg/day × 2 days, 20 mg/day × 2 days, 10 mg/day × 2 days
6	Piperacillin/tazobactam, vancomycin, fluconazole	Midazolam, fentanyl, insulin, lactulose, levothyroxine, norepinephrine, pantoprazole	Hydrocortisone 50 mg every 6 hours × 1 day
7	Piperacillin/tazobactam	Midazolam, alprazolam, fentanyl, insulin, famotidine, heparin	
8	Vancomycin	Midazolam, fentanyl, insulin, famotidine, heparin, simvastatin, gabapentin, venlafaxine, calcium gluconate	Hydrocortisone 50 mg every 8 hours × 8 days
9		Midazolam, lorazepam, omeprazole, ursodiol, clonidine, levetiracetam, ondansetron, metaclopramide, hydralazine, heparin	
10	Piperacillin/tazobactam, vancomycin	Midazolam, famotidine, aspirin, clopidogrel, insulin, heparin, metaclopramide, docusate	Prednisone 20 mg/day × 3 days, Hydrocortisone 100 mg every 8 hours × 2 days
11		Midazolam, protonix, amitryptyline, bupropion, carvedilol, clonazepam, folic acid, acetaminophen, digoxin, ondansetron, heparin, tacrolimus	Methylprednisolone 60 mg every 12 hours × 3 days
12	Vancomycin, clindamycin, tamiflu	Midazolam, fentanyl, morphine, heparin, insulin, pantoprazole, bumetanide	Methylprednisolone 60 mg/day × 4 days, prednisone 40 mg/day × 3 days
13		Midazolam, enoxaparin, famotidine, insulin, metoprolol, aspirin, hydralazine, lisinopril, simvastatin	
14	Vancomycin, levofloxacin, flagyl, aztreonam	Midazolam, fentanyl, heparin	Methylprednisolone 60 mg every 12 hours × 6 days
15	Vancomycin	Propofol, omeprazole, heparin, amlodipine	
16		Midazolam, fentanyl, protonix, ondansetron, darbepoetin, folic acid, cyanocobalamin, hydralazine, amldipine, lisinopril	
17	Vancomycin, levofloxacin	Midazolam, fentanyl, protonix, insulin, lactulose, levothyroxine, sertraline, hydralazine, gabapentin, carvedilol	
19	Vancomycin, piperacillin/tazobactam, levofloxacin	Heparin, hydralazine, labetalol, metoprolol, levetiracetam, lorazepam, omeprazole, phenytoin	Prednisone 20 mg/day × 2 days
20	Vancomycin	Midazolam, morphine, amlodipine, labetalol, metoprolol, protonix, phenytoin, heparin	
21	Vancomycin, tobramycin, ampicillin/sulbactam	Midazolam, fentanyl, heparin, ibuprofen, amiodarone, metoprolol, omeprazole	
22	Vancomycin, levofloxacin	Famotidine, heparin, metaclopramide, diphenhydramine	
23	Vancomycin, doripenem, colistin	Bumetanide, midazolam, ascorbic acid, famotidine, insulin, vitamin A, zinc, aripiprazole, escitalopram, heparin	
24		Paroxetine, pramipexole, simvastatin, omeprazole, heparin, midazolam, insulin, metoprolol, bumetanide	
25		Midazolam, heparin, omeprazole, haloperidol, olanzapine, insulin	Prednisone 40 mg/day × 6 days
26		Famotidine, darbepoetin, amlodipine, labetolol, folic acid, thiamine, dexmedetomidine, heparin	
27	Doripenem, doxycycline, micafungin, flagyl	Midazolam, fentanyl, promethazine, pantoprazole, levetiracetam, propofol, lactulose	Prednisone 50 mg every 8 hours × 8 days
28		Midazolam, haloperidol, omeprazole, levothyroxine, heparin, insulin, furosemide	
29	Doripenem, tobramycin, fluconazole, erythromycin	Morphine, ondansetron, rifaximin, pantoprazole, lactulose, insulin, aspirin, heparin, levetriacetam, midazolam, phenytoin, vasopressin	
30		Aspirin, benztropine, famotidine, gabapentin, hydrochlorothiazide, metoclopramide, miralax, insulin, heparin, valproic acid, docusate	Methylprednisolone 125 mg every 6 hours × 2 days
31		Azathioprine, bumetanide, clopidogrel, ezetimibe, famotidine, fentanyl, furosemide, heparin, metoprolol, midazolam, nitroglycerin patch, omeprazole, provastatin	Methylprednisilone 125 mg every 6 hours × 3 days, 40 mg every 6 hours × 2 days, prednisone 60 mg/day × 5 days
32	Vancomycin, gentamycin, aztreonam	Midazolam, fentanyl, aspirin, carbemazepam, haloperidol, metoprolol, omeprazole, phenytoin, insulin, heparin	Methylprednisolone 60 mg every 6 hours × 6 days
33	Linezolid	Midazolam, lorazepam, fentanyl, aspirin, digoxin, insulin, metoprolol, famotidine, heparin	
34	Vancomycin, piperacillin/tazobactam, micofungin	Midazolam, fentanyl, insulin, aspirin, amlodipine, metaclopramide, famotidine, heparin	Methylprednisolone 100 mg every 12 hours × 9 days
35	Vancomycin, piperacillin/tazobactam, micofungin, colistin, bactrim	Foscarnate, pantoprazole, diphenhydramine, insulin, fludrocortisone, levothyroxine, ursodiol	Methylprednisolone 10 mg × 1 day
36	Vancomycin, meraopenum, valgancyclovir, cefipime, dapsone, levofloxacin	Ondansetron, omeprazole, metoprolol, mycophenolate mofetil, sildenafil	Prednisone 25 mg/day × 250 days
37	Piperacillin/tazobactam, vancomycin, levofloxacin	Midazolam, fentanyl, aspirin, heparin, omeprazole, simvastatin	Methylprednisolone 40 mg/day × 2 days, prednisone 40 mg/day × 1 day, prednisone 20 mg/day × 3 days
38	Flagyl, levofloxacin, aztreonam	Midazolam, fentanyl, morphine, norepinephrine, vasopressin, famotidine, heparin	
39	Doripenam, colistimethate, vancomycin	Midazolam, fentanyl, lortab, zolpidem, metoprolol, omeprazole, heparin	
40	Vancomycin, piperacillin/tazobactam, levofloxacin	Midazolam, fentanyl, metaclopramide, aspirin, azathioprine, clopidogrel, furosemide, ondansetron, simvastatin, famotidine, heparin	Prednisone 20 mg/day × 7 days
41	Vancomycin, piperacillin/tazobactam, clindamycin	Midazolam, fentanyl, propofol, insulin, omeprazole	
42	Vancomycin, cefipime, levofloxacin, acyclovir	Midazolam, fentanyl, ondansetron, pantoprazole, heparin	
44		Midazolam, fentanyl, haloperidol, insulin, famotidine, heparin	Methylprednisolone 125 mg every 6 hours × 2 days
45		Midazolam, fentanyl, ondansetron, insulin, omeprazole, heparin	Hydrocortisone 100 mg every 8 hours × 3 days
46	Linezolid, piperacillin/tazobactam, tobramycin	Midazolam, Diltiazem, mycophenolate mofetil, tacrolimus, famotidine, insulin, heparin	Hydrocortisone 100 mg every 8 hours × 6 days, prednisone 40 mg/day × 3 days
47	Vancomycin, cefipime, levofloxacin	Midazolam, aspirin, captopril, furosemide, simvastatin, insulin	Prednisone 40 mg/day × 3 days, 30 mg/day × 2 days
49		Midazolam, aspirin, atorvastatin, bisoprolol, clopidogrel, fluticasone, folic acid, pantoprazole, heparin	Methylprednisolone 60 mg every 6 hours × 4 days, 40 mg every 12 hours × 2 days, prednisone 40 mg/day × 1 day
50	Vancomycin, aztreonam, tobramycin, daptomycin	Midazolam, hydroxyzine, darbepoetin, levothyroxine, ferrous sulfate, ergocalciferol, pancrelipase, omeprazole	Hydrocortisone 20 mg every 12 hours × 37 days
51	Vancomycin, piperacillin/tazobactam, doripenam, fluconazaole	Midazolam, fentanyl, furosemide, pancrelipase, magnesium oxide, famotidine, insulin, heparin	
52	Vancomycin, piperacillin/tazobactam, daptomycin	Midazolam, fentanyl, levothyroxine, darbepoetin, fluticasone, lactulose, paroxetime, insulin, heparin	Hydrocortisone 100 mg every 8 hours × 5 days
53	Tamiflu, piperacillin/tazobactam, cefipime, daptomycin, doripenam	Midazolam, darbepoetin, bumetanide, famotidine, insulin, heparin	
54	Vancomycin, piperacillin/tazobactam	Morphine, oxycodone, furosemide, famotidine, insulin, heparin	Hydrocortisone 50 mg every 6 hours × 3 days
55	Vancomycin, piperacillin/tazobactam, levofloxacin, daptomycin	Ferrous sulfate, folic acid, levothyroxine, metaclopramide, pravastatin, digoxin tacrolimus, omeprazole, heparin	
56	Daptomycin, linezolid, tobramycin, colistin, zithromax	Midazolam, fentanyl, dexmedetomidine, omeprazole, heparin	
57	Vancomycin, piperacillin/tazobactam, levofloxacin, tamiflu	Midazolam, fentanyl, clonazepam, gabapentin, famotidine, insulin, heparin	
58	Piperacillin/tazobactam	Midazolam, morphine, furosemide, metoprolol, citalopram, pantoprazole, aspirin, acetazolamide, insulin, heparin	Methylprednisolone 60 mg every 8 hours × 4 days, prednisone 60 mg/day × 5 days, 40 mg/day × 5 days, 30 mg/day × 5 days
59	Vancomycin, cefipime, levofloxacin	Midazolam, fentanyl, dopamine, norepinephrine, simvastatin, aspirin, omeprazole, heparin	Hydrocortisone 100 mg every 8 hours × 3 days, 50 mg every 8 hours × 2 day, 50 mg every 12 hours × 7 days
60	Vancomycin, cefipime, tobramycin	Midazolam, fentanyl, norepinephrine, pantoprazole, heparin	

^a^Data not included for Subjects 18, 43, and 48 because transdiaphragmatic twitch pressure measurements not obtained due to anatomic constraint.

**Figure 3:**
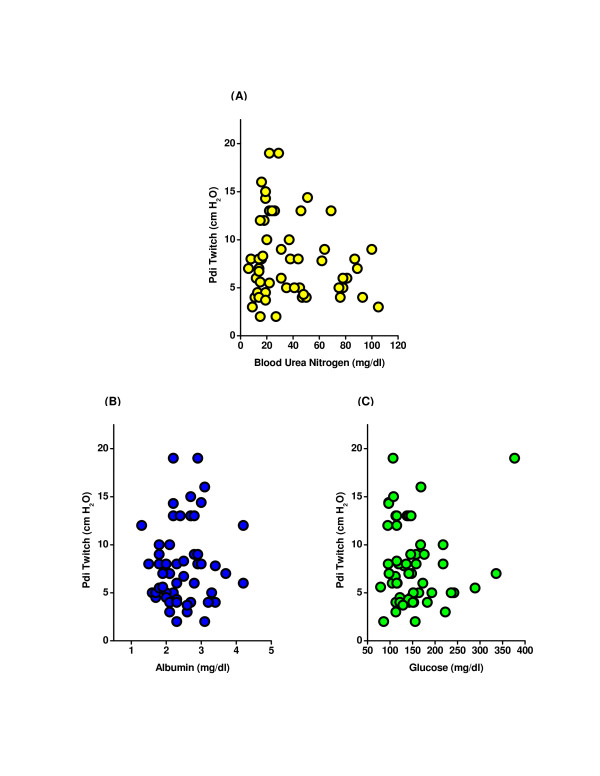
**Correlation of transdiaphragmatic twitch pressure to blood urea nitrogen, albumin, and glucose levels**. Transdiaphragmatic twitch pressure (PdiTw) as a function of **(A) **blood urea nitrogen (BUN), **(B) **albumin, and **(C) **glucose levels. There was no significant correlation between any these parameters and PdiTw. Specifically, correlation coefficients and *P *values for regression of PdiTw to parameters were, respectively, 0.146 and 0.277 for BUN, 0.072 and 0.596 for albumin, and 0.032 and 0.815 for glucose levels (all nonsignificant).

**Figure 4:**
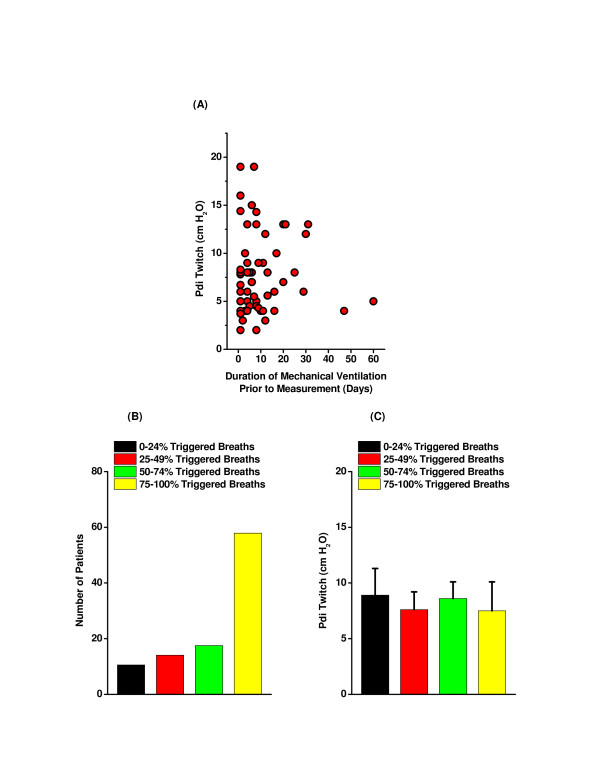
**Relationship of prior duration of mechanical ventilation and ventilator triggering to diaphragm strength**. **(A) **Transdiaphragmatic twitch pressure (PdiTw) as a function of the duration of mechanical ventilation prior to measurement of PdiTw. There was no statistically significant correlation of PdiTw to duration of ventilation prior to measurement, with a correlation coefficient of 0.020 and *P *= 0.881 for this assessment (nonsignificant). **(B) **The majority of subjects actively initiated (that is, triggered) ventilator breaths more than 75% of the time. **(C) **The level of diaphragm strength (PdiTw) did not correlate with the level of triggering, with the same PdiTw observed at all triggering levels.

We also found that there was no significant correlation between PdiTw and either BUN, albumin, or glucose levels (Figures 3A, B, C). While many patients were receiving steroids (regimens provided in Table 3), we found no correlation between steroid dosage and PdiTw values (see Additional file 4). In addition, we found no relationship between the number of days subjects had been on mechanical ventilation prior to testing and the level of PdiTw (Figure 4A). This finding contrasts with recent reports suggesting that patients on mechanical ventilation for longer durations have progressively lower levels of diaphragm strength [6,18]. One potential explanation for this difference is that the patients examined in the present study were all ventilated with assist modes of mechanical ventilation, while previous work that demonstrated a strong relationship between mechanical ventilation and the development of diaphragm weakness specifically restricted examination to patients who were on controlled mechanical ventilation with little or no spontaneous respiratory activity [18]. As shown in Figure 4B, our patient population had a high level of spontaneous respiratory activity, with the majority of patients triggering more than 75% of ventilator breaths. Of interest, we also found that PdiTw was similar over the range of levels of ventilator triggering observed in the present study (Figure 4C).

### Relationship of diaphragm strength to patient outcomes

**Figure 5:**
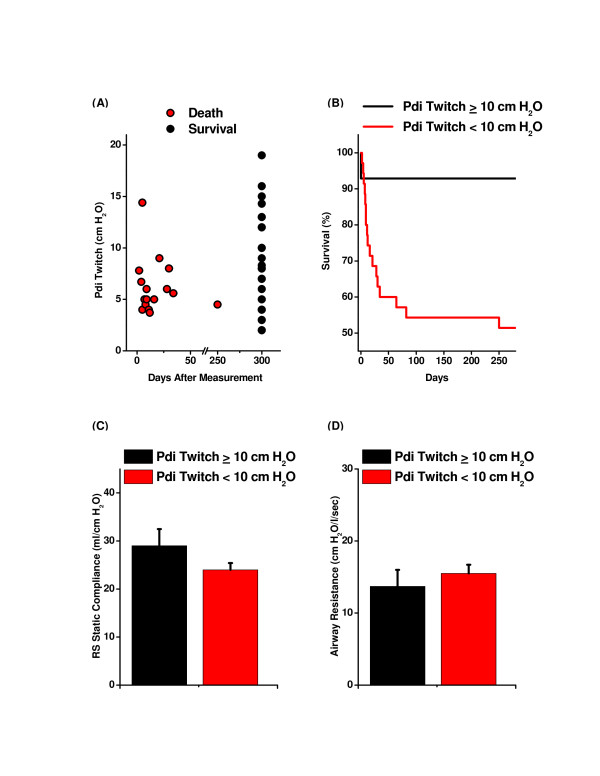
**Relationship of diaphragm strength to survival**. **(A) **Survival of patients (days after measurement, *x *axis) as a function of transdiaphragmatic twitch pressure (PdiTw) level (*y *axis). Patients that died had low average PdiTw levels (6.3 ± 0.6 cmH_2_O) while survivors had higher PdiTw levels (8.9 ± 0.9 cmH_2_O, *P *= 0.044). **(B) **Survival curves for subjects with PdiTw ≥10 cmH_2_O (*n *= 15) and PdiTw <10 cmH_2_O (*n *= 42). Weak subjects had a significantly higher mortality (49%) than strong subjects (7%, *P *= 0.022). To exclude the possibility that the greater mortality in the weakest patients may have been due to the presence of more severe lung dysfunction, we also examined **(C) **respiratory system (RS) static compliance and **(D) **airway resistance. There was no significant difference in RS static compliance or airway resistance for patients with PdiTw ≥10 cmH_2_O and PdiTw <10 cmH_2_O, indicating that the greater mortality ion the weakest patients was not due to concomitant lung dysfunction.

To assess the relationship between diaphragm strength and mortality, we plotted PdiTw against patient days of survival (Figure 5A). Patients that died were significantly weaker than survivors, with PdiTw averaging 6.3 ± 0.6 and 8.9 ± 0.9 cmH_2_O, respectively, for these two groups (*P *< 0.04). To further analyze this relationship, we used Fisher exact testing and ROC curve analyses to determine the level of PdiTw that best discriminated between survival and mortality [17]. Both forms of testing found this boundary to be 10 cmH_2_O. Patients with PdiTw ≥10 cmH_2_O had only a 7% mortality (one death out of 14 patients) while patients with PdiTw <10 cmH_2_O had a 49% mortality (17 deaths out of 35 patients, *P *= 0.022 for comparison of the two groups; Figure 5B). Because indices of lung function may influence mortality, we also compared respiratory system static compliance and airway resistance between patients with PdiTw ≥10 cmH_2_O and patients with Pdi <10 cmH_2_O (Figures 5C, D). Lung mechanics were not significantly different between these two groups of patients, indicating that level of diaphragm function, not lung function, best correlated with survival in our patients. In addition, patients with PdiTw ≥10 cmH_2_O and PdiTw <10 cmH_2_O had similar Sequential Organ Failure Assessment scores (7.6 ± 0.6 and 6.9 ± 0.4, respectively) and Charlson Comorbidity Indices (2.7 ± 0.5 and 2.5 ± 0.3, respectively).

We also evaluated the possible mechanism(s) by which diaphragm weakness may have influenced the incidence of death. In this cohort, five of the patients with PdiTw <10 cmH_2_O that died were receiving vasopressors when care was withdrawn; vasopressors and mechanical ventilation were stopped simultaneously in these patients and death occurred as a result of combined respiratory failure and hypotension. In the remaining 12 patients with PdiTw <10 cmH_2_O that died, none met criteria for brain death, all maintained motor drive to the respiratory pump, none were on vasopressors, and the only form of continuous life support that these patients were receiving was mechanical ventilation. Prior weaning trials had been attempted and all 12 patients had failed to reach extubation criteria. Death occurred in these 12 patients when mechanical ventilation was withdrawn. These data suggest that the presence of severe diaphragm weakness limited weaning trial success in these 12 patients and may have influenced the decision to withdraw care.

**Figure 6:**
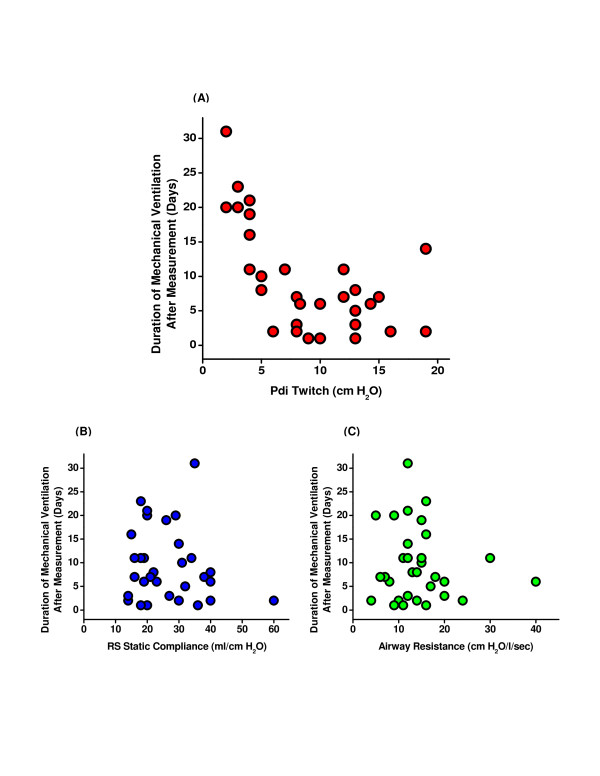
**Relationship of diaphragm strength to ventilator weaning duration**. **(A) **Duration of mechanical ventilation after measurement of transdiaphragmatic twitch pressure (PdiTw) as a function of the level of PdiTw; each symbol represents data from a single subject. Patients with PdiTw ≥10 cmH_2_O required significantly shorter times to wean from mechanical ventilation when compared with patients with PdiTw <10 cmH_2_O (*P *= 0.016). The time required to wean from mechanical ventilation bore no relationship, however, to **(B) **the respiratory system (RS) static compliance or **(C) **the airway resistance.

With respect to other outcome measures, seven patients with PdiTw <10 cmH_2_O were transferred to a long-term ventilator facility while only one of the patients with PdiTw ≥10 cmH_2_O was transferred to a LTAC facility. In addition, the time required to wean survivors from mechanical ventilation was a function of PdiTw, with time to wean increasing significantly for patients with PdiTw <10 cmH_2_O (Figure 6A). On average, duration of mechanical ventilation after PdiTw measurements was 12.3 ± 1.7 days for patients with PdiTw <10 cmH_2_O but only 5.5 ± 2.0 days for patients with PdiTw ≥10 cmH_2_O (*P *= 0.016). In contrast, duration of mechanical ventilation had no relationship to either respiratory system static compliance (Figure 6B) or airway resistance (Figure 6C).

## Discussion

The present study indicates that diaphragm weakness is a significant determinant for poor outcomes in mechanically ventilated MICU patients We found that the incidence of death was 49% in the patients with the weakest diaphragms (that is, with PdiTw <10 cmH_2_O) but was only 7% for patients with PdiTw levels ≥10 cmH_2_O. One possible explanation for the far greater mortality in patients with PdiTw <10 cmH_2_O could be that weakness is simply a marker for multiorgan system failure and that damage to these other organs was primarily responsible for patient deaths. Surprisingly, however, we found that indices of disease severity (for example, lung mechanics, Sequential Organ Failure Assessment scores, Charlson Comorbidity Indexes) were almost identical in patients with PdiTw levels ≥10 cmH_2_O and in patients with PdiTw <10 cmH_2_O, suggesting that the relationship between diaphragm weakness and mortality is not simply an epiphenomenon. Moreover, the majority of patient deaths (12 of 18 deaths) were the direct result of withdrawal of mechanical ventilatory support in weak patients and, in each case, occurred after unsuccessful weaning attempts. Weakness almost certainly contributed to the inability to wean these patients from mechanical ventilation and thereby may have influenced the decision to withdraw care.

We also found that a high percentage of patients with PdiTw <10 cmH_2_O required transfer to LTAC units. Reports indicate that long-term outcomes for this group of patients are poor, with a high percentage (51%) dying within 1 year [19]. As a result, the high rate of transfer of weak patients to these units represents a poor outcome. In addition, we found that the relationship between diaphragm strength and duration of mechanical ventilation for patients that did not die and remained in the ICU was curvilinear, with duration increasing progressively as PdiTw levels fell to lower values (Figure 6A). Weak patients with PdiTw <10 cmH_2_O required more than twice as long to wean from mechanical ventilation than stronger patients with PdiTw levels ≥10 cmH_2_O. Moreover, the duration of mechanical ventilation did not correlate with the level of lung dysfunction but only with the level of diaphragm strength.

We also evaluated our data to ascertain the role of infections, BUN, albumin and glucose levels in the induction of diaphragm weakness in mechanically ventilated patients. We found the level of diaphragm weakness in mechanically ventilated MICU patients did not correlate with BUN, glucose, or albumin levels despite previous reports associating these factors with prolonged mechanical ventilation [11-13]. In contrast, we found that evidence of infection was a predictor of strikingly lower levels of diaphragm strength than that observed for non-infected patients. This finding is consistent with multiple previous animal studies demonstrating that infection rapidly reduces diaphragm force generation, decreases diaphragm mitochondrial function, activates diaphragm proteolytic pathways, and reduces diaphragm contractile protein function [9,20-26].

While infected patients had the weakest diaphragms, even the non-infected mechanically ventilated patients in our study had a median level of PdiTw (13 cmH_2_O), which is substantially lower than that observed for normal healthy adults (30 cmH_2_O). There are at least two potential explanations for the weakness observed in the non-infected patients. First, many of the patients in our study were chronically ill, with multiple comorbid conditions including heart failure, malignancy, liver and renal diseases. Each of these entities has negative effects on muscle function, and it is possible that the pre-intubation muscle function of these patients may have been appreciably lower than that observed in normal subjects.

In addition, use of mechanical ventilation can result in diaphragm inactivity and atrophy [27-30]. Numerous animal studies have provided evidence of this phenomenon, and, more recently, several elegant studies indicate that loss of diaphragm function occurs in patients who are subjected to controlled mechanical ventilation with minimal or no spontaneous respirations [18,27]. Our patients were all ventilated using assisted modes of mechanical ventilation and may therefore have had less inactivity-induced diaphragm dysfunction than observed in patient populations ventilated with controlled modes of mechanical ventilation. Nevertheless, it is still possible that ventilator-induced inactivity contributed to the level of diaphragm weakness observed in our non-infected patients. As a corollary, the level of weakness observed in our infected patients may represent the combined effects of chronic illness, ventilator-induced inactivity, and infection-induced diaphragm dysfunction.

One should note, however, that the non-infected patients had a level of PdiTw (median of 13 cmH_2_O) that was sufficiently high for this group of patients to be expected to have good outcomes (that is, a low death rate and an average wean time from mechanical ventilation of about 5 days) according to the data presented in Figures 5 and 6. Only the infected mechanically ventilated patients, as a group, had low enough PdiTw levels (median of 5.5 cmH_2_O) to expect poor outcomes (that is, a high mortality and a protracted need for mechanical ventilation). These data therefore argue that even if all of the diaphragm weakness observed in our non-infected patients was a consequence of ventilator-induced inactivity, this level of weakness alone would not be expected to result in poor patient outcomes. Our data would instead suggest that only the combination of ventilator-induced inactivity and infection may produce sufficient diaphragm weakness to negatively influence patient survival and duration of mechanical ventilation.

## Conclusions

In summary, we found that mechanically ventilated MICU patients have severe diaphragm weakness, that the clinicians caring for these patients greatly underestimate the severity of diaphragm weakness present, and that infections are a major risk factor for the development of diaphragm weakness in this population. We also found that diaphragm weakness was associated with poor patient outcomes, including a significantly increased mortality, an increased transfer to LTACs and a markedly longer duration required for weaning from mechanical ventilation.

Diaphragm weakness appears to be a major risk factor for respiratory failure and death in mechanically ventilated MICU patients; theoretically, pharmacological treatments that improve diaphragm strength should reduce the duration of mechanical ventilation and MICU mortality. Currently no such agents are used in clinical practice, but recent experimental studies indicate that pharmacological inhibition of selected cellular pathways can prevent diaphragm weakness in animal models of critical illness [31,32]. There is an urgent need to translate these pharmacological treatments from the bench to the bedside in order to prevent or reverse diaphragm weakness in mechanically ventilated MICU patients. Such therapies are likely to influence both acute and long-term outcomes.

## Key messages

• Recent work indicates that many mechanically ventilated MICU patients have severe diaphragm weakness, but the causes and consequences of this weakness remain controversial.

• The present study indicates that infection is a major risk factor for development of diaphragm weakness in MICU patients treated with assist modes of mechanical ventilation.

• This work also demonstrates that the level of diaphragm weakness is a novel predictor of clinical outcomes; the weakest patients have a high mortality and require prolonged durations of mechanical ventilation.

• This study indicates that clinicians underestimate the severity of diaphragm dysfunction in mechanically ventilated critically ill patients.

## Abbreviations

BUN: blood urea nitrogen; LTAC: long-term acute care; MICU: medical ICU; PdiTw: transdiaphragmatic twitch pressure; PEEP: positive end-expiratory pressure.

## Competing interests

The authors declare that they have no competing interests.

## Authors' contributions

GSS drafted the protocol, performed the measurements, analyzed the pressure tracings, obtained patient data, interpreted the data, and drafted and revised the final manuscript. LAC assisted in performing the measurements, obtaining patient data, and had a major impact on the interpretation of the data and revision of the manuscript. Both authors read and approved the final manuscript.

## Supplementary Material

Additional file 1 **presents additional methods**. Click here for file

Additional file 2 **Table S1 presenting the demographic and clinical data in 57 subjects who underwent PdiTw measurements**. Click here for file

Additional file 3 **Figure S1. Relationship of PdiTw values to PEEP levels**. PdiTw values are shown as a function of PEEP levels. We found no correlation between the level of PEEP and PdiTw measurements. The correlation coefficient and p value for regression of PdiTw to PEEP were 0.093 and 0.492, respectively (NS). Click here for file

Additional file 4 **Figure S2. Effect of Steroids on PdiTw values**. Cumulative dosages of steroids was calculated and converted into hydrocortisone equivalents, then plotted as a function of PdiTw. As shown, steroid dosage had no relationship to measured PdiTw. The correlation coefficient and p value for regression of PdiTw to hydrocortisone equivalents were 0.064 and 0.637, respectively (NS). Click here for file
