# Supplementary material for: Diaphragm weakness in mechanically ventilated critically ill patients
Source: Crit Care. 2013 Jun 20;17(3):R120. doi: 10.1186/cc12792 (PMC3840677; doi:10.1186/cc12792)
Supplement: Additional file 1 — presents additional methods. [file cc12792-S1.DOCX]

**Additional file 1: Methods**

**Survey of Attending Physician’s Assessment of Diaphragm Strength**

The attending physician caring for each subject was asked to complete the following questionnaire:

**Please circle the statement that best describes your patient.**

**I estimate that my patient’s respiratory muscle strength is:**

a) Normal (Pdi Twitch more than 30 cm H_2_O)

b) Slightly reduced (Pdi Twitch between 26 and 30 cm H_2_O)

c) Moderately reduced (PdiTwitch between 16 and 25 cm H_2_O)

d) Severely reduced (Pdi Twitch between 6 and 15 cm H_2_O)

e) Profoundly reduced (Pdi Twitch less than 5 cm H_2_O)
