# Supplementary material for: Diaphragm weakness in mechanically ventilated critically ill patients
Source: Crit Care. 2013 Jun 20;17(3):R120. doi: 10.1186/cc12792 (PMC3840677; doi:10.1186/cc12792)
Supplement: Additional file 2 — Table S1 presenting the demographic and clinical data in 57 subjects who underwent PdiTw measurements. [file cc12792-S2.DOCX]

**TABLE S1. Demographic and Clinical Data in 57 Subjects* Who Underwent PdiTw Measurements**

| **Subject #** | **Age (years)** | **Gender** | **BMI** | **Reason for MICU Admission** | **Other Diagnoses** | **Mode of MV** | **MV Days Before PdiTw Measured** | **Total ICU Days** | **Outcome** |
| --- | --- | --- | --- | --- | --- | --- | --- | --- | --- |
| **1** | 56 | M | 25.5 | CVA | Respiratory failure, renal failure, respiratory arrest | AC | 1 | 6 | Died |
| **2** | 27 | F | 33.1 | Sepsis | Respiratory failure, anemia, liver abscess, renal failure | AC | 1 | 15 | Survived |
| **3** | 51 | M | 37.7 | Carbon Monoxide Inhalation | Respiratory failure, hypertension, acute MI | PRVC | 4 | 11 | Survived |
| **4** | 76 | F | 40 | GI bleed | Respiratory failure, PNA, hypernatremia, hypercalcemia | PRVC | 1 | 9 | Survived |
| **5** | 56 | F | 35.3 | Respiratory failure | CVA, BOOP | PRVC | 12 | 25 | Survived |
| **6** | 61 | F | 27.2 | Cholangitis | Respiratory failure, cholelithiasis, pancreatitis, cirrhosis | PC | 3 | 8 | Died |
| **7** | 67 | F | 32.3 | Respiratory failure | COPD, sepsis, atrial fibrillation, PNA, renal failure, anemia | PS | 12 | 35 | Survived |
| **8** | 55 | F | 34 | Respiratory failure | Sepsis, COPD, DM, CVD, paraplegia, ulcerative colitis | PRVC | 8 | 47 | Survived |
| **9** | 28 | F | 13.9 | Acetaminophen overdose | Respiratory failure, ALI, hepatitis C, liver failure, ESRD, encephalopathy, neuroblastoma, malnutrition | PS | 20 | 23 | Survived |
| **10** | 76 | M | 19.6 | Respiratory failure | Sepsis, COPD, PNA | PRVC | 16 | 35 | LTAC |
| **11** | 38 | F | 38.5 | Respiratory failure | Eosinophilic PNA, renal transplant | PC | 1 | 4 | Survived |
| **12** | 49 | M | 41.1 | Respiratory failure | ARDS, influenza, bacteremia, pulmonary edema, DM, sinusitis | PC | 20 | 21 | LTAC |
| **13** | 58 | M | 45.4 | Respiratory failure | CHF, DM, anoxic brain injury, HTN | AC | 7 | 22 | Survived |
| **14** | 50 | M | 47.7 | Septic shock | Sepsis, abdominal abscess, COPD, renal failure | PRVC | 25 | 89 | Died |
| **15** | 40 | M | 22.7 | Septic shock | Respiratory failure, endocarditis, seizure, hepatitis C, renal failure, HTN | PRVC | 6 | 19 | Survived |
| **16** | 46 | F | 24.6 | Respiratory failure | MI, history of GI bleed, IV drug use, history of endocarditis | PRVC | 17 | 23 | Survived |
| **17** | 68 | M | 31.6 | Encephalopathy | Respiratory failure, hepatic cancer, metabolic acidosis, DM, cirrhosis, hepatitis C | PRVC | 1 | 3 | Died |
| **19** | 56 | M | 42.6 | Respiratory failure | PNA, DM, anoxic brain injury, HTN, COPD, seizure | PRVC | 10 | 27 | Survived |
| **20** | 40 | M | 22.7 | Obtundation | Respiratory failure, Staph endocarditis, hepatitis C, HTN, renal failure, depression, seizure | PRVC | 6 | 18 | Survived |
| **21** | 63 | M | 32.2 | Septic shock | Respiratory failure, Ludwigs angina, neck abscess, PNA, DVT | PRVC | 13 | 20 | Survived |
| **22** | 73 | M | 19.4 | Respiratory failure | Previous polio, weakness, scoliosis, bacteremia | PRVC | 2 | 23 | Survived |
| **23** | 52 | M | 49.7 | Renal failure | Respiratory failure, COPD, remote history of GBS, renal failure, DM, atrial fibrillation, HTN, CAD | AC | 4 | 8 | Survived |
| **24** | 84 | F | 27.5 | Respiratory failure | Asthma, COPD, DM, HTN | PRVC | 8 | 11 | Survived |
| **25** | 68 | M | 40.8 | Respiratory failure | Lung cancer, obesity, anemia | PRVC | 21 | 30 | Survived |
| **26** | 36 | M | 36.4 | Respiratory failure | Pancreatitis, PRES | PRVC | 4 | 10 | Survived |
| **27** | 33 | F | 29.7 | Respiratory failure | Subphrenic abscess, ascites | PC | 11 | 54 | LTAC |
| **28** | 59 | F | 13.4 | Respiratory failure | Sepsis, COPD, pulmonary emboli, PNA | PRVC | 31 | 34 | LTAC |
| **29** | 55 | M | 51.8 | Respiratory failure | Pulmonary HTN, PNA, pleural effusion, OSA | PRVC | 8 | 17 | Died |
| **30** | 66 | F | 36 | Cardiac arrest | Respiratory failure, PNA, COPD, CHF, atrial fibrillation | AC | 8 | 15 | Survived |
| **31** | 61 | M | 24.7 | Respiratory failure | Pulmonary fibrosis, atrial fibrillation | PC | 1 | 4 | Survived |
| **32** | 63 | F | 29.2 | Respiratory failure | Hepatitis C, atrial fibrillation, sepsis | PRVC | 9 | 30 | Died |
| **33** | 76 | F | 20.5 | Respiratory failure | CAD, CHF, atrial fibrillation, mitral stenosis, COPD | PS | 8 | 16 | Died |
| **34** | 62 | F | 37.6 | Respiratory failure | HTN, CAD, DM, OSA | PRVC | 4 | 20 | Died |
| **35** | 60 | F | 32.8 | Respiratory failure | HLH, C.diff colitis, renal failure, candidemia, MOF | PRVC | 60 | 67 | Died |
| **36** | 63 | F | 17.9 | Respiratory failure | Double lung transplant, scleroderma | PRVC | 5 | 250 | Died |
| **37** | 74 | F | 30.6 | Respiratory failure | COPD, MI, cellulitis | PRVC | 1 | 14 | LTAC |
| **38** | 55 | F | 18.4 | Shock | Respiratory failure, ARDS, liver abscess | PRVC | 6 | 10 | Survived |
| **39** | 42 | M | 28.1 | Respiratory failure | Bacteremia, burns (35% BSA) | PC | 47 | 63 | Survived |
| **40** | 61 | M | 24.4 | Respiratory failure | PNA, IPF, renal failure | PC | 1 | 29 | Died |
| **41** | 28 | F | 24.8 | Respiratory failure | Sepsis, ATN, shock liver, CHF | PRVC | 5 | 8 | Survived |
| **42** | 22 | M | 18.4 | Sepsis | Osteomyelitis, MRSA bacteremia, abscess | PRVC | 1 | 22 | Survived |
| **44** | 39 | M | 29.9 | Respiratory failure | ARDS, pulmonary emboli, hyperglycemia | PRVC | 3 | 5 | Survived |
| **45** | 45 | F | 22.7 | Respiratory failure | SVT, DVT, dysphagia | PC | 6 | 14 | Survived |
| **46** | 76 | M | 41.5 | Respiratory failure | Lung transplant, PNA, renal failure, anemia | PS | 4 | 25 | Survived |
| **47** | 71 | M | 25.5 | Respiratory failure | PNA, critical illness myopathy, DM, CAD, prostate mass | PS | 7 | 42 | LTAC |
| **49** | 48 | F | 32.6 | Respiratory failure | Pulmonary fibrosis | PC | 1 | 5 | Died |
| **50** | 48 | F | 21.6 | Septic shock | PNA, empyema, DM, ESRD, adrenal insufficiency | AC | 30 | 41 | Survived |
| **51** | 46 | F | 37.7 | Respiratory failure | Anoxic encephalopathy, pancreatitis, ARDS, bacteremia | PC | 4 | 34 | Died |
| **52** | 47 | M | 37.6 | Respiratory failure | ESRD, DM, HTN, PNA, atrial fibrillation | PRVC | 16 | 25 | Died |
| **53** | 32 | F | 22.7 | Respiratory failure | ARDS, PNA, renal injury, H1N1, anemia | PC | 29 | 36 | LTAC |
| **54** | 68 | M | 18.9 | Respiratory failure | Renal failure, COPD, CMML, laryngeal CA, PNA, cardiomyopathy | PS | 9 | 56 | LTAC |
| **55** | 76 | M | 41.5 | Respiratory failure | Lung transplant, PNA, bronchiolitis | PRVC | 13 | 47 | Died |
| **56** | 23 | F | 16 | Respiratory failure | CF, MRSA PNA, malnutrition | PRVC | 6 | 88 | Died |
| **57** | 46 | F | 25.1 | Respiratory failure | PNA, elevated troponin, renal failure, nephrolithiasis | PRVC | 1 | 7 | Survived |
| **58** | 37 | F | 19.3 | Respiratory failure | COPD, septic shock, atrial flutter, NSTEMI, renal injury, UTI | PRVC | 11 | 30 | Survived |
| **59** | 85 | F | 30.2 | Respiratory failure | Sepsis, renal failure, DM, anemia, NSTEMI, hypernatremia | AC | 1 | 12 | Died |
| **60** | 73 | M | 23.1 | Respiratory failure | COPD, anoxic encephalopathy, renal failure | PRVC | 1 | 13 | Died |

* Data not included for subjects 18, 43, and 48 because PdiTw measurements not obtained due to anatomic constraints

Abbreviations: AC = assist control, ALI = acute lung injury, ARDS = adult respiratory distress syndrome, ATN= acute tubular necrosis, BMI = body mass index, BOOP = bronchiolitis obliterans with organizing pneumonia, CA=cancer, CAD= coronary artery disease, CF = cystic fibrosis, CHF = congestive heart failure, CMML = chronic myelomonocytic leukemia, COPD = chronic obstructive pulmonary disease, CVA = cerebrovascular accident, CVD = cerebrovascular disease, DM = diabetes mellitus, DVT = deep vein thrombosis, ESRD = end stage renal disease, GBS = Guillain-Barré syndrome, GI = gastrointestinal, HLH = Hemophagocytic lymphohistiocytosis, HTN = hypertension, IPF = idiopathic pulmonary fibrosis, IV = intravenous, LTAC = long term acute care facility, MI = myocardial infarction, MICU = medical intensive care unit, MOF = multi-organ failure, MRSA = methicillin resistant Staphylococcal aureus, MV = mechanical ventilation, NSTEMI = non-ST segment elevation myocardial infarction, OSA = obstructive sleep apnea, PC = pressure control, PNA = pneumonia, PRES = Posterior reversible encephalopathy syndrome, PRVC = pressure regulated volume control, PS = pressure support, SVT = supraventricular tachycardia, UTI = urinary tract infection
