# Supplementary material for: Diaphragm weakness in mechanically ventilated critically ill patients
Source: Crit Care. 2013 Jun 20;17(3):R120. doi: 10.1186/cc12792 (PMC3840677; doi:10.1186/cc12792)
Supplement: Supplementary file 3 [file cc12792-S3.DOCX]

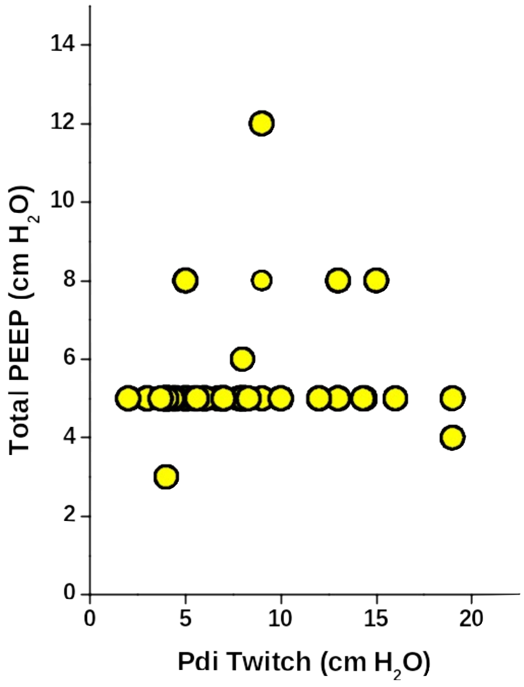

Total PEEP (cm H_2O)
0
2
4
6
8
10
12
14
0
5
10
15
20
Pdi Twitch (cm H_2O)
