# Supplementary material for: Diaphragm weakness in mechanically ventilated critically ill patients
Source: Crit Care. 2013 Jun 20;17(3):R120. doi: 10.1186/cc12792 (PMC3840677; doi:10.1186/cc12792)
Supplement: Supplementary file 4 [file cc12792-S4.DOCX]

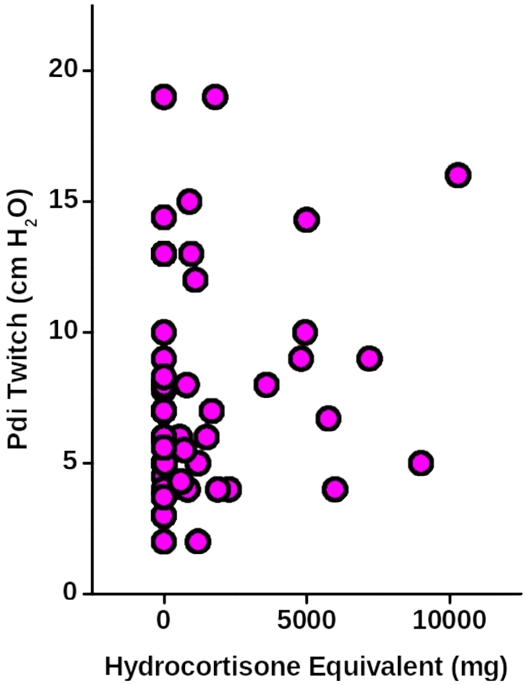

20
15
10
5
0
Pdi Twitch (cm H2O)
0
5000
10000
Hydrocortisone Equivalent (mg)
